# Expression of the Bovine NK-Lysin Gene Family and Activity against Respiratory Pathogens

**DOI:** 10.1371/journal.pone.0158882

**Published:** 2016-07-13

**Authors:** Junfeng Chen, Chingyuan Yang, Polyana C. Tizioto, Huan Huang, Mi O. K. Lee, Harold R. Payne, Sara D. Lawhon, Friedhelm Schroeder, Jeremy F. Taylor, James E. Womack

**Affiliations:** 1 Department of Veterinary Pathobiology, College of Veterinary Medicine, Texas A&M University, College Station, United States of America; 2 Embrapa Southeast Livestock, São Carlos, Brazil; 3 Division of Animal Sciences, University of Missouri, Columbia, United States of America; 4 Department of Physiology and Pharmacology, College of Veterinary Medicine, Texas A&M University, College Station, United States of America; ContraFect Corporation, UNITED STATES

## Abstract

Unlike the genomes of many mammals that have a single *NK-lysin* gene, the cattle genome contains a family of four genes, one of which is expressed preferentially in the lung. In this study, we compared the expression of the four bovine *NK-lysin* genes in healthy animals to animals challenged with pathogens known to be associated with bovine respiratory disease (BRD) using transcriptome sequencing (RNA-seq). The expression of several *NK-lysins*, especially *NK2C*, was elevated in challenged relative to control animals. The effects of synthetic peptides corresponding to functional region helices 2 and 3 of each gene product were tested on both model membranes and bio-membranes. Circular dichroism spectroscopy indicated that these peptides adopted a more helical secondary structure upon binding to an anionic model membrane and liposome leakage assays suggested that these peptides disrupt membranes. Bacterial killing assays further confirmed the antimicrobial effects of these peptides on BRD-associated bacteria, including both *Pasteurella multocida* and *Mannhemia haemolytica* and an ultrastructural examination of *NK-lysin*-treated *P*. *multocida* cells by transmission electron microscopy revealed the lysis of target membranes. These studies demonstrate that the expanded bovine *NK-lysin* gene family is potentially important in host defense against pathogens involved in bovine respiratory disease.

## Introduction

Cationic antimicrobial peptides (AMPs) are important molecules in the host innate immune system and are widespread in both plants and animals [1]. One of the conserved characteristics of AMPs is their cationic and hydrophobic composition, which makes them potent killers of microbial targets with cytoplasmic membranes rich in anionic phospholipids and they are selectively safe to host cells with neutral charged membranes. Several mechanisms have been proposed to describe the AMP-target interaction, and the basic steps are similar [2]. AMP molecules are attracted to targets by the electrostatic interaction between the cationic residues and anionic phospholipids in target membranes and adopt an amphipathic structure, with the hydrophobic face interacting with the hydrophobic lipid bilayers and the hydrophilic face interacting with the anionic head groups of phospholipids. Unlike antibiotics, which can induce the development of resistance in microbes within a short application period and cause potential threats to public health [3], the electrostatic interaction between cationic AMPs and anionic target membranes reduces the development of resistance while preserving the efficacy of antimicrobial effects. Therefore, AMPs are candidates for the development of new antimicrobial drugs.

Human *granulysin* and porcine *NK-lysin* are AMPs secreted from cytotoxic T and NK cells [4, 5]. Both molecules and their derivatives are active against a broad spectrum of microorganisms including bacteria, fungi, viruses and also cancer cells [6–9]. One of the most interesting of their antimicrobial activities is their capacity to directly kill extracellular *Mycobacterium tuberculosis*, which is particularly resistant to the human immune response [10, 11]. They also exhibit potent effects on intracellular *Mycobacterium tuberculosis* following permeation of the cellular membrane by the pore-forming protein perforin [12]. We previously reported that a single copy of the *NK-lysin* gene in many mammals has expanded to create a gene family with four expressed members in cattle, *NK1*, *NK2A*, *NK2B* and *NK2C* [13]. *NK2A*, *NK2B* and *NK2C* arose by tandem segmental duplication and share high sequence identity with each other, while *NK1* is more diverged. Four synthetic peptides spanning helices 2 and 3 of each gene product display antimicrobial activities against both gram-positive *Staphylococcus aureus* and gram-negative *Escherichia coli*. Three of the bovine *NK-lysins* are highly expressed in intestinal Peyer’s patch, which is consistent with the expression of its human and pig orthologs. However, *NK2C* exhibits a distinct expression profile, being most highly expressed in lung which indicates that it may potentially have a novel function in the bovine respiratory system.

Bovine respiratory disease (BRD) or shipping fever is the most common infectious disease affecting both the upper and lower respiratory tracts of cattle and is a major cause of economic loss in North America through treatment costs, reduced performance and mortalities [14–16]. BRD is multi-factorial with a variety of stressors, including host factors (age, genetics and host immunity) [17–19], environmental factors (temperature, transport, commingling and ventilation) [20–22] and pathogens (bacteria and viruses) leading to disease. Several microorganisms have been implicated in the pathogenesis of BRD including bacterial agents, such as *Mannheimia haemolytica* [23, 24], *Pasteurella multocida* [23], *Mycoplasma bovis* [25] and *Histophilus somni* [26], and viral agents, such as *bovine viral diarrhea virus* (BVDV) [25], *bovine respiratory syncytial virus* (BRSV) [27], *bovine herpesvirus-1* (BHV-1 or IBR) [27] and *bovine parainfluenza-3 virus* (PI-3) [28]. Interactions between environmental stressors and infectious agents are critical to the development of BRD. Environmental factors (such as transport or weaning) weaken the host’s immune system and predispose animals to viral infections, which then facilitate secondary infections by bacterial pathogens, which lead to the onset of BRD. Many strategies have been proposed to prevent and treat BRD, including feedlot management to reduce environmental stresses, vaccination of animals to improve immune responses, breeding of cattle that are resistant to BRD pathogens [29] and anti-microbial agents (antibiotics and sulfas) to treat infected cattle.

The identification of genes that influence the host’s response to pathogens is an important step towards identifying the specific genetic variants which could be used in breeding cattle with an increased resistance to infections. The aim of this study was to investigate the potential roles of four bovine *NK-lysin* genes in host response to BRD associated pathogens. By comparing the read depths of each *NK-lysin* family member from whole transcriptome sequencing data, we found that the expression of *NK2C* in lung was elevated in animals that had been challenged with multiple pathogens associated with BRD. All four peptides synthesized in the previous study not only exhibited disruptive effects on negatively charged model membranes, but also showed antimicrobial activities against *P*. *multocida* and *M*. *haemolytica*. These results suggest that the bovine *NK-lysin* genes, especially *NK2C*, are potentially important in the host’s immune response to the pathogens contributing to respiratory diseases. Further studies will be beneficial in identifying genetic variants in the *NK-lysin* gene family that might be associated with differential disease susceptibility.

## Results

### Elevated Expression of Bovine NK2C in Pathogen Challenged Animals

**Fig 1:**
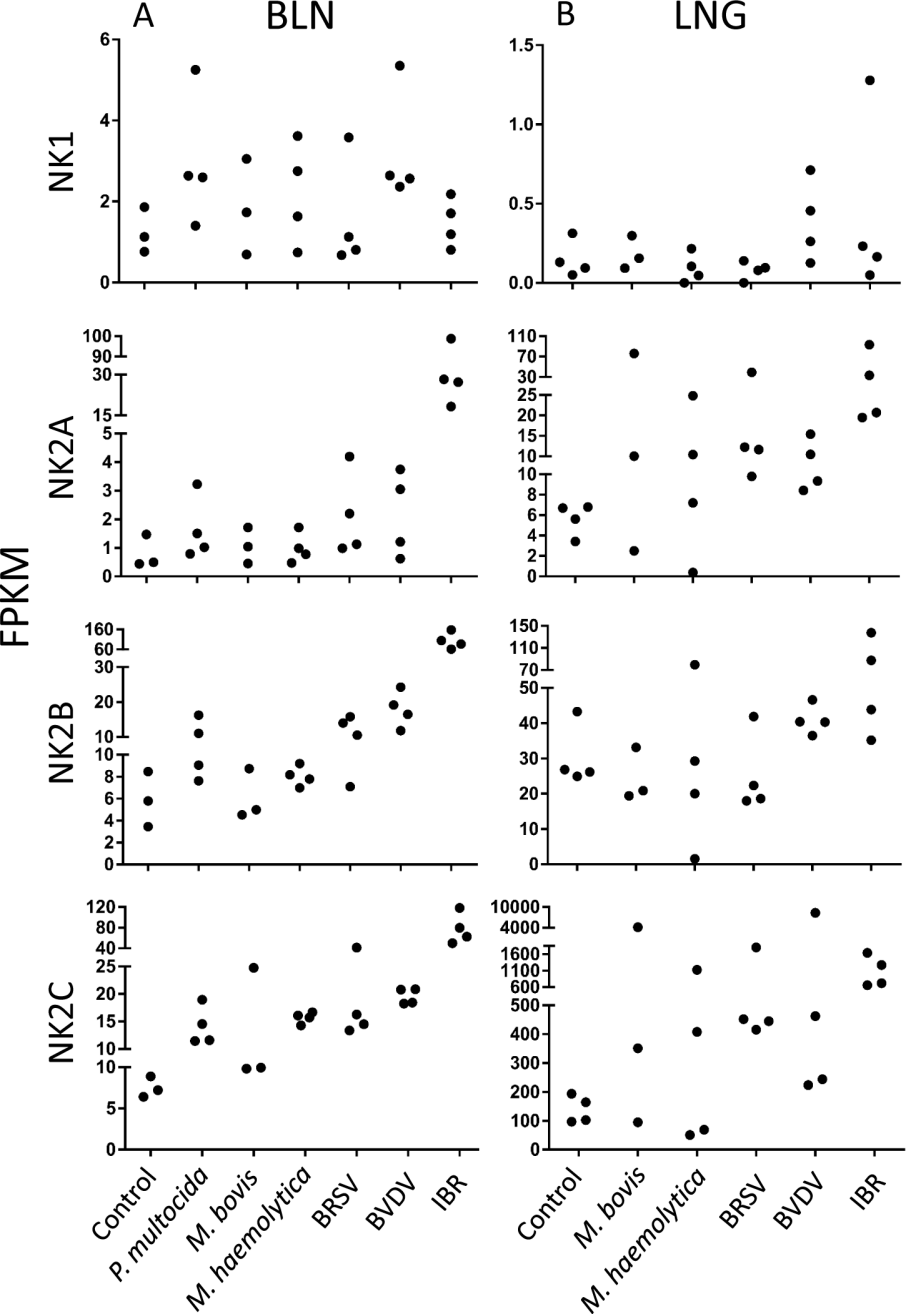
Expression of four bovine *NK-lysin* genes in bronchial lymph node (BLN, left panel) and lung (LNG, right panel) among healthy animals and animals challenged with *P*.*multocida*, *M*.*bovis*, *M*.*haemolytica*, BRSV, BVDV and IBR. The Y axis shows the FPKM value, and each black dot represents the FPKM value of an individual. Three or four individuals were included in each control and challenged group.

To investigate the potential contributions of the bovine *NK-lysin*s to host resistance to BRD associated pathogens, we compared RNA-seq FPKM values for each *NK-lysin* gene in both bronchial lymph node and lung lesion tissues from healthy animals and animals challenged with a set of BRD-related pathogens [30, 31]. Generally, the expression of *NK1* was very low in these tissues while *NK2C* exhibited relatively high expression in both tissues. Furthermore, expression of *NK2C* in the lungs of most of the challenged animals was significantly higher than for the other three genes (Fig 1). When animals were challenged with the IBR virus, the expression of *NK2A*, *NK2B* and *NK2C* was significantly elevated in bronchial lymph nodes, and an increased expression of *NK2B* and *NK2C* in bronchial lymph nodes was also observed in most of the animals challenged with other pathogens (Fig 1). Overall, an elevated expression of *NK2A* and *NK2C* was observed in most of the pathogen challenged animals. In contrast to the similar expression levels in the four healthy control animals, the expression of *NK2C* was elevated by > 20-fold in two of the experimentally challenged animals.

### Secondary Structural Changes of Bovine NK-Lysin Peptides upon Liposome Binding

**Fig 2:**
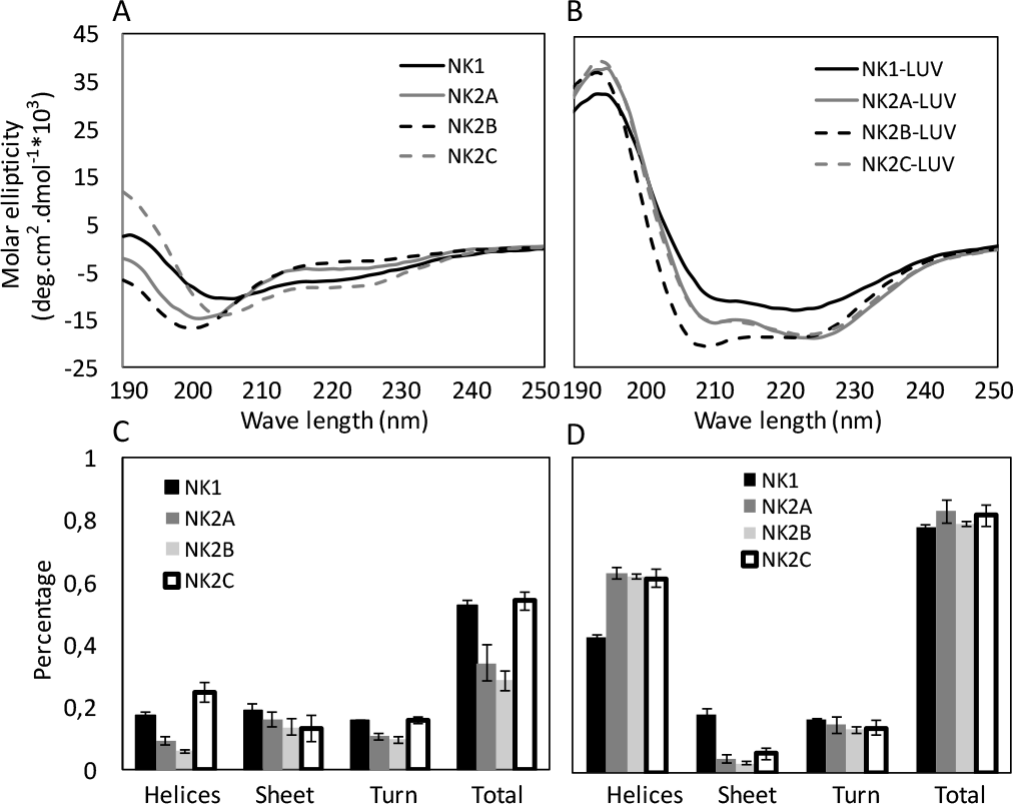
Secondary structural changes of the four synthetic bovine *NK-lysin* peptides upon liposome binding. CD spectra of *NK-lysin* peptides in lipid-free (A) and lipid-bound states (B) are compared. Estimated secondary structural contents, including alpha-helices, beta-sheet, beta-turn and the total secondary structure in lipid-free and lipid-bound states are shown in (C) and (D), respectively.

To investigate the interactions between bovine *NK-lysin* peptides and bio-membranes in target microorganisms, we employed circular dichroism (CD) spectroscopy to study the potential conformational changes of these peptides upon their interaction with anionic liposome mimicking bacterial membranes. The CD spectrum of each of the peptides in buffer presented a single negative band at 200 nm, which indicated an unordered structure (random coil) (Fig 2A). However, two negative bands at 208 nm and 222 nm along with a positive band at 192 nm were exhibited when mixed with the negatively charged liposome (35% POPE + 50% POPG + 15% Cardiolipin), suggesting the conformational transition of the peptides from random coils to a more ordered structure (Fig 2B). The proportional contents of the alpha–helix, beta-sheet and beta-turn of each peptide in both lipid-free and lipid-bound states were also compared (Fig 2C and 2D). The proportions of the total ordered secondary structures, especially the alpha helices, were enhanced in the presence of liposome for all peptides. The fractions of each secondary structure for *NK2A*, *NK2B* and *NK2C* were comparable upon interaction with liposome, while those for *NK1* were different with a lower degree of helicity and a higher proportion of beta-sheet in the lipid-bound state. This result was consistent with the behavior of most cationic AMPs, which exhibit an unordered structure in aqueous solution but adopt a more helical conformation upon interaction with anionic phospholipid membranes [32].

### Bovine NK-Lysin Peptides Disrupt Model Membranes

**Fig 3:**
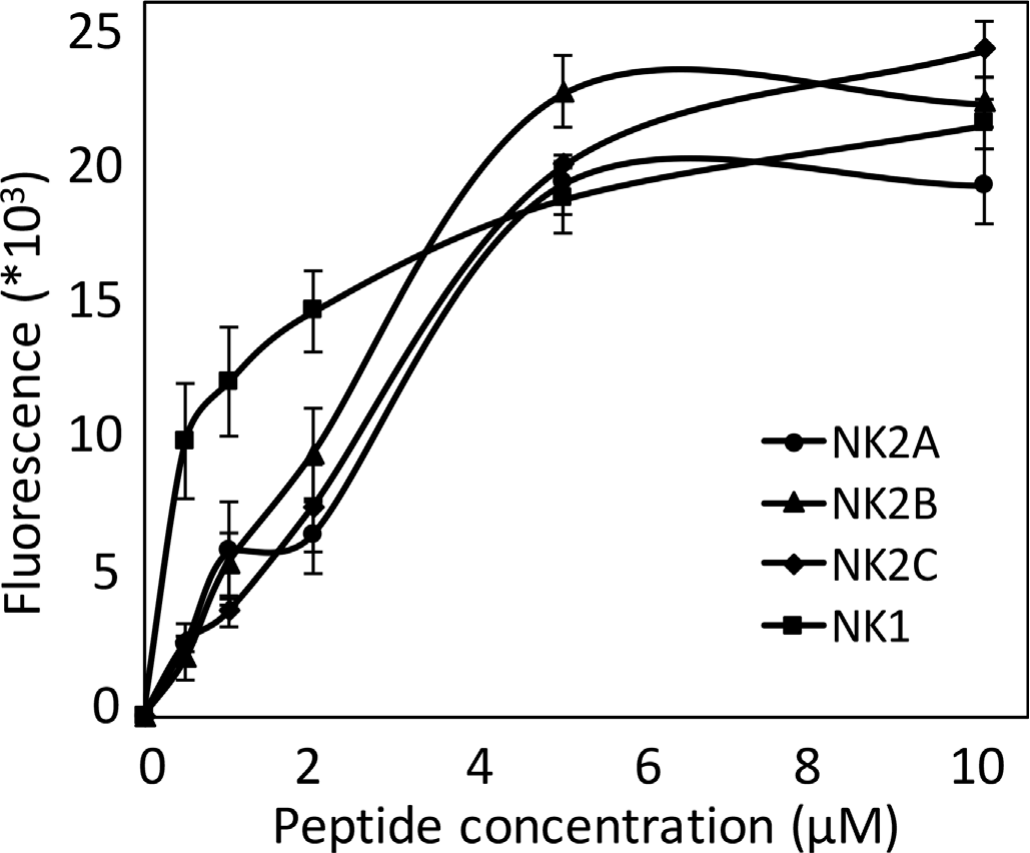
Intensities of released fluorescent dye from liposome plotted against concentration of bovine *NK-lysin* peptides.

A liposome leakage assay was performed to investigate the influence of the synthetic bovine *NK-lysin* peptides on a model membrane. The peptides began to disrupt the liposome at a concentration of 0.5 μM, resulting in the release of entrapped fluorescent dye (Fig 3). As the concentrations were increased to 1 μM and subsequently to 2 μM, the released fluorescence intensities were correspondingly elevated and the leakage of entrapped dye caused by *NK1* peptide was remarkably greater than that caused by the other peptides. However, the leakage detected by fluorescence for the four peptides was comparable at concentrations of 5 μM, and was maintained at this level when the concentration was increased to 10 μM, indicating the complete disruption of the vesicles at a peptide concentration of 5 μM.

### Bovine NK-Lysin Peptides Exhibit Antimicrobial Effects on BRD-Causing Bacteria *P*. *multocida* and *M*. *haemolytica*

**Fig 4:**
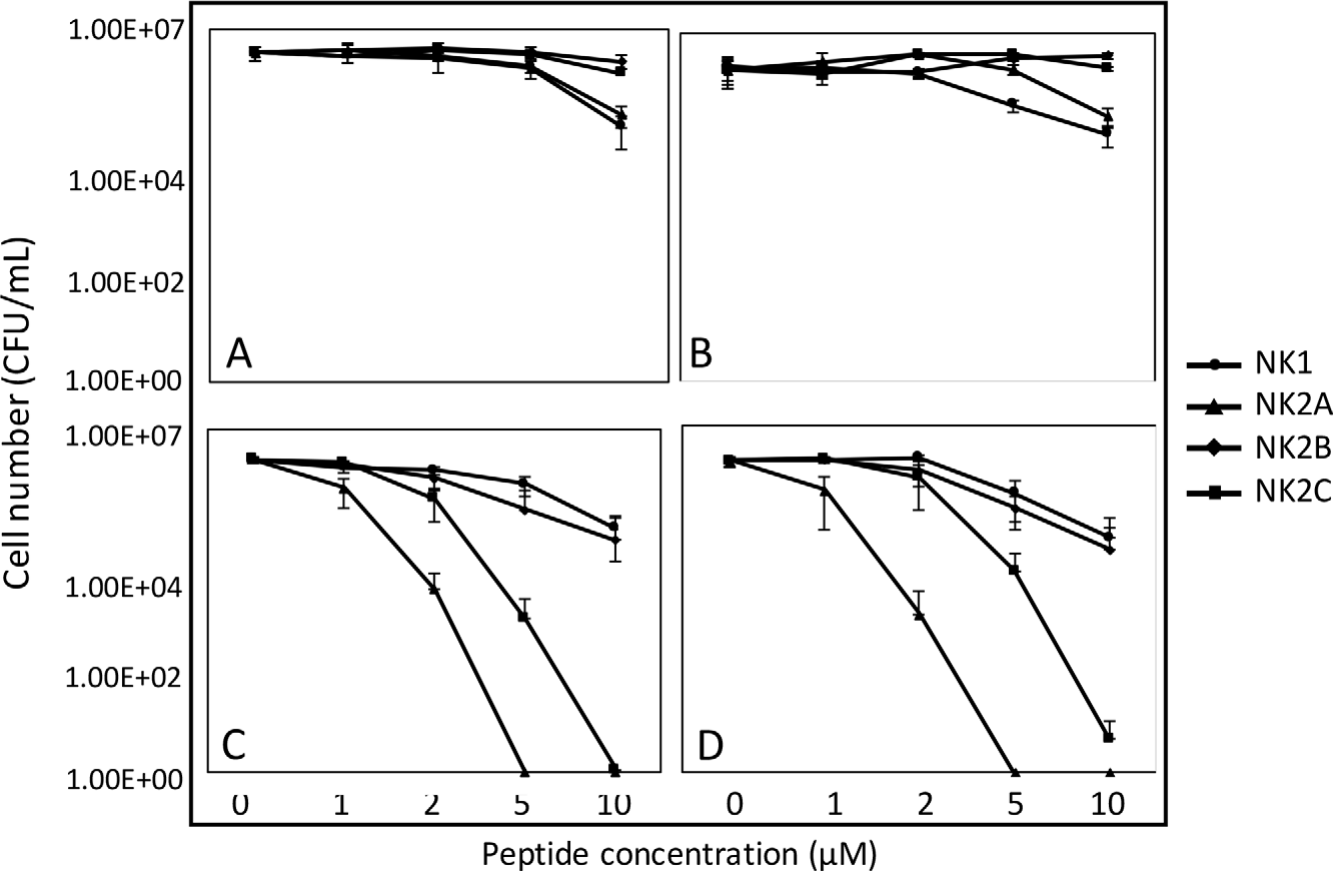
**Antimicrobial effects of bovine *NK-lysin* peptides on BRD-causing pathogens *P*. *multocida* strains ATCC 43019 (A) and ATCC 43137 (B), *M*. *haemolytica* strains ATCC BAA-410 (C) and ATCC 33396 (D).** Surviving cell numbers after peptide treatment are shown on the Y axis. Error bars represent the standard deviations calculated from four biological replications.

The antimicrobial activities of bovine *NK-lysin* peptides were tested against two *P*. *multocida* bacterial strains (ATCC 43019 and ATCC 43137) and two *M*. *haemolytica* bacterial strains (ATCC BAA-410 and ATCC 33396). Overall, the *P*. *multocida* strains were less susceptible to the peptides (Fig 4A and 4B). Significant cell number losses were not observed until the peptide concentration was increased to 10 μM for *NK1* and *NK2A* when an approximately 50-fold decrease in viable cells was produced. The *NK2B* and *NK2C* peptides did not display obvious killing abilities. In contrast, the *NK2A* and *NK2C* peptides displayed potent antimicrobial activities against both *M*. *haemolytica* strains in a dose-dependent manner (Fig 4C and 4D). An approximately 5-fold decrease in cell numbers resulted from incubation with 1 μM of *NK2A* for 1 h, and the complete elimination of *M*. *haemolytica* cells was achieved with 5 μM of *NK2A* or 10 μM of *NK2C*. *NK1* and *NK2B* peptides exhibited weaker killing abilities against *M*. *haemolytica* and achieved an approximately 50-fold cell loss at the highest concentration of 10 μM. Surprisingly, *M*. *haemolytica* cells were susceptible to the *NK2A* peptide but resistant to the *NK1*, which was the most potent peptide against *P*. *multocida* as well as *E*. *coli* and *S*. *aureus* in our previous study.

### Bovine NK-Lysin Peptide Lyses Cell Membranes

**Fig 5:**
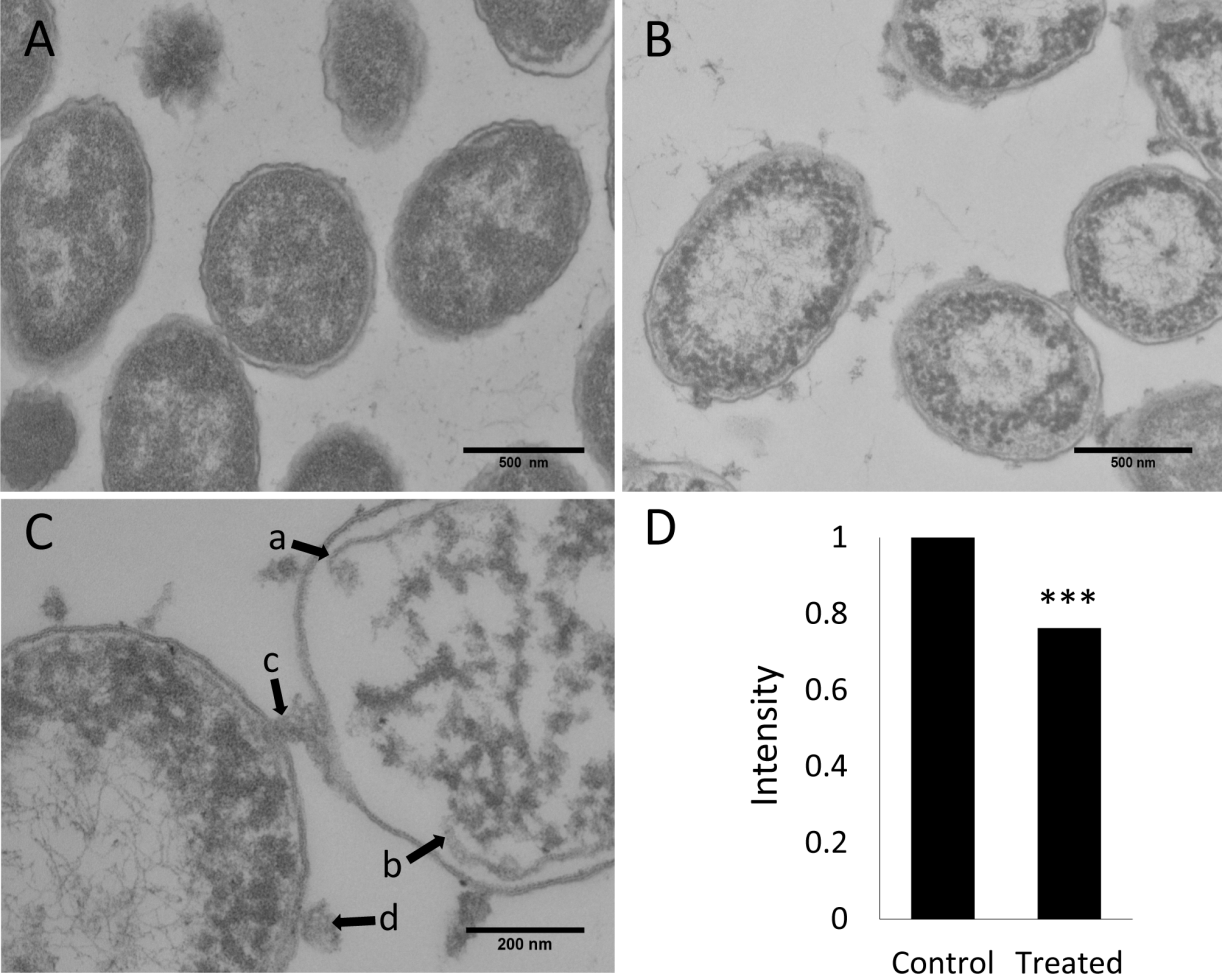
Influence of 20 μM of bovine *NK1* peptide on the cell membrane of *P*. *multocida* (ATCC 43019) examined by transmission electron microscopy. A) Control cells. (B) and (C) Cells treated with 20 μM *NK1* peptide for 30 mins. (D) Statistical analysis of the average electron intensity of control cells versus *NK1*-treated cells. Thirty cells from each group were used for statistical analysis.

The impacts of bovine *NK-lysin* peptides on the cell morphology and membrane integrity of *P*.*multocida* cells were examined by transmission electron microscopy (TEM) (Fig 5). The untreated cells displayed intact outer and inner membranes with a clear periplasmic space, and the cytoplasm was homogeneously filled with electron dense material (Fig 5A). Although the cell morphology was maintained, severe cellular damage with large clear zones in the cytoplasm indicating the leakage of cytoplasmic contents was observed when cells were treated with 20 μM of *NK1* peptide for 30 mins (Fig 5B). In addition, cytoplasmic constituents were coagulated into non-membrane-enclosed bodies within the areas near membranes. *NK1* peptide treatment also caused rupture of the cytoplasmic membrane (Fig 5C arrows a & b) that resulted in leakage of the cytoplasmic contents and the release of intracellular material that attached in aggregates on the exterior of the cell (Fig 5C arrows c & d). Statistical analysis revealed that the overall electron density of an untreated *P*. *multocida* cell was significantly higher than that of a cell treated with bovine *NK1* peptide for 30 mins, suggesting the leakage of cytoplasmic contents in *NK1*-treated cells. Therefore, bovine *NK1* peptides were shown to cause the release of cytoplasmic material from a *P*. *multocida* cell by damaging its cell membrane, eventually leading to cell death and the appearance of empty “shells” (ghost cells).

## Materials and Methods

### RNA-Seq Analysis

RNA-seq data were generated and analyzed at the University of Missouri. Computations were performed on the HPC resources at the University of Missouri Bioinformatics Consortium (UMBC). Animal challenge and whole transcriptome sequencing protocols were previously described [30, 31]. The study was carried out in strict accordance with the NIH Guide for the Care and Use of Laboratory Animals as described in protocol #16424, approved by the University of California, Davis Institutional Animal Care and Use Committee. Briefly, the steers were produced by mating Angus sires to advanced generation Angus-Hereford crossbred dams at the University of California Davis Sierra Field Station located in Brown's Valley, CA. Blood was collected and steers seronegative, or with the lowest titers against each bacterial and viral pathogen, were selected and the steers had not been vaccinated against any BRD pathogens. The six to eight month old steers were transported to the University of California, Davis, where they were maintained in pens, fed a 65% concentrate starter diet and water were provided *ad libitum*. The challenge studies were performed sequentially starting with the control animals and with animals housed in groups by control or pathogen challenge. In this study, we analyzed the bovine *NK-lysin* expression in both the lung lesion and bronchial lymph node tissues [31] collected from the same individual. Since the four bovine *NK-lysin*s share high sequence identity, especially *NK2A*, *NK2B* and *NK2C*, protocols were designed with extra care to remap the short (2 x 50 bp) reads specifically to each gene. Basically, all short reads from each sample were mapped allowing no mismatches to an index built with the mRNA sequences of all four *NK-lysin*s using Bowtie 2 [33]. The mapping quality which measures the degree of confidence in the mapping of a read to a specific single locus was used to assess whether the reads were uniquely mapped to one of the four genes, and the number of these uniquely mapped reads was counted for each *NK-lysin* gene. Quality trimmed reads with a size of < 25 bp were excluded from this analysis.

### Peptide Synthesis

Four 30-aa peptides corresponding to the functional region helices 2 and 3 of each gene product were synthesized with > 95% purity by Peptide 2.0 Inc (Chantilly, VA). Lyophilized peptides were dissolved and aliquoted in phosphate-buffered saline (PBS) (pH 7.4) and stored at −20°C before use. Concentrations of the stock peptides were determined by amino acid assay in the Texas A & M University Protein Chemistry Lab.

### Circular Dichroism Assay

Phospholipids POPE, POPG and Cardiolipin were purchased from Avanti Polar Lipids (Alabaster, AL). Lyophilized lipids were dissolved in chloroform to a concentration of 20 mg/mL and stored at −20°C before use. To prepare the negatively charged liposome containing 35% POPE, 50% POPG and 15% cardiolipin, the appropriate amounts of the lipid stock solutions were mixed and the chloroform was evaporated under N_2_ with constant rotation and the solution was further dried in a vacuum environment overnight. The dried mixture was re-suspended in potassium phosphate buffer (10 mM, pH 7.4) to a concentration of 10 mM, bath-sonicated for fifteen mins and subjected to five freeze-thaw cycles. The solution was subsequently extruded through a polycarbonate membrane (100 nm), back and forth, twenty times and stored at 4°C before use. The CD spectrum was obtained in the same phosphate buffer containing 20 μM of each peptide with or without liposome at a working concentration of 1 mM at room temperature with a JASCO J-815 CD Spectrometer (JASCO, Easton, MD). Each sample was scanned five times at wavelengths ranging from 190 to 250 nm with the step resolution of 1 nm. All data were expressed as the mean molar ellipticity (deg.cm^2^.dmol^-1^), background (buffer or liposome only) subtracted and the content of each secondary structure including alpha-helix, beta-sheet and beta-turn was estimated with the analysis software provided by the manufacturer of the CD spectrometer using CONTIN with SDP48 as the reference set.

### Liposome Leakage Assay (Fluorescence Quenching Assay)

Liposome containing 35% POPE, 50% POPG and 15% cardiolipin and the entrapped fluorophore/quencher (ANTS/DPX) dye pair were prepared by a method similar to that described above, except that the potassium phosphate buffer was replaced by a dye-containing Pipes buffer (5 mM ANTS/50 mM DPX/20 mM Pipes/27.5 mM NaCl, pH 7.4) to suspend the dried lipids. The liposome with entrapped ANTS/DPX was subjected to a G-50 Sephadex chromatography column to eliminate the free dye, and the total lipid concentration of the collected dye-free fractions was determined by a phosphorus assay [34]. Dye-free liposome was mixed with or without peptides in a Pipes buffer (20 mM Pipes/ 85 mM NaCl, pH 7.4) to a final lipid concentration of 300 μM and peptides at a serial dilutions of 0.5, 1, 2, 5 and 10 μM. The fluorescence intensity was measured using a BioTek Synergy 2 microplate reader, with excitation filter 330/80, emission filter 540/35. Fluorescence intensity was measured before and after the addition of peptides.

### Antimicrobial Killing Assay

Overnight cultures of four pathogenic bacterial strains (*P*. *multocida* ATCC 43019, ATCC 43137 and *M*. *haemolytica* ATCC BAA-410, ATCC 33396) were sub-cultured in brain-heart infusion medium at 37°C for an additional 2.5 hours to mid-exponential phase, washed and re-suspended in PBS (pH 7.4) to a cell concentration of 5×10^6^ CFU/ml. A 100-μl aliquot of cells was incubated with 20 μl PBS buffer or buffer plus each *NK-lysin* peptide prepared in the same buffer to the final working concentrations of 1, 2, 5 and 10 μM at 37°C for 1 h. After the 1 h incubation, a 100-μl aliquot of each mixture was diluted in PBS buffer to an approximate cell concentration of 3×10^3^ CFU/ml, from which another 100-μl aliquot was plated on trypticase soy agar plates supplemented with 5% sheep blood. Colonies of the surviving cells were manually counted after overnight incubation at 37°C in a 5% carbon dioxide atmosphere. Experiments were performed with four biological replicates and repeated twice. Data provided are from a single experiment.

### Transmission Electron Microscopy

50-μl overnight culture of *P*. *multocida* ATCC 43019 was sub-cultured in 5 ml BHI medium for 2 h. Four ml of the culture were subsequently washed and re-suspended into PBS buffer, and incubated with 20 μM *NK1* peptide or an equal volume of PBS buffer for 30 mins at 37°C. The mixture was fixed with an equal volume of 3% glutaraldehyde and samples for TEM examination were prepared following the previously described protocol [13]. Briefly, the samples were osmicated, *en bloc* stained with uranyl acetate, dehydrated in ethanol, and embedded in epoxy resin. Thin sections were prepared and EM images of the cells were recorded with a Morgagni 268 TEM (FEI, Hillsboro, OR). Student *t*-test (paired, two-tailed, unequal variances) was performed to compare the mean electron intensities of thirty cells from both the control and NK-lysin-treated groups.

## Discussion

Several factors have been suggested to influence the antimicrobial capacities of AMPs, including the net positive charge, hydrophobicity and amphipathicity. Increased positive charge and hydrophobicity are major contributors to the enhancement of the antimicrobial effects of AMPs [35, 36]. The net charges (pH = 7) and hydrophobicities (pH = 6.8) differ among the functional regions of the four examined *NK-lysin* peptides, with *NK1* possessing the highest hydrophobicity and largest hydrophobic face with the least positive charge and *NK2A* being the most positively charged peptide. Bacterial killing assays revealed that *NK1* exhibited the highest antimicrobial effects on *E*. *coli*, *S*. *aureus* and *P*. *multocida* while *NK2A* was the most potent peptide against *M*. *haemolytica*. During gene family expansion, each paralog has evolved to encode a peptide with specific antimicrobial properties, which has enabled the activity of the bovine *NK-lysin* family against a broad range of microbes.

Several studies have been undertaken to search for genes and associated genetic variants that contribute to host resistance to respiratory pathogens or responses to vaccines, and candidate genes or genomic regions now include the *MHC* region, *TLRs*, *PVRL1* and *DST* [29, 37]. With the application of high density SNP genotyping technology, genome-wide association studies have become a preferred method for identifying genetic markers linked to phenotypic variation in host response [29, 38, 39]. Another effective approach to the identification of genetic variants that could be beneficial to animal breeding is a candidate gene approach based on the known biological functions of gene products. Since innate immunity is not only an essential component of the host’s immune response but also affects subsequent acquired immunity, genes that are expressed in the innate immune system are strong candidates for their effects on host resistance to infectious agents. Human *NK-lysin* is an effector molecule in the innate immune system, and its expression is induced by antigenic stimulation indicating its potential role in host responses to antigens [40]. Despite the existence of large individual variation in expression within individuals challenged with the same BRD-associated pathogen, the expression of bovine *NK-lysin* genes, especially *NK2C*, in both the bronchial lymph node and lung were elevated in most of the challenged animals. The synthetic peptides corresponding to the functional helices 2 and 3 of each gene product also exhibited antimicrobial effects on the BRD-associated bacterial microbes, *P*. *multocida* and *M*. *haemolytica*, and antimycobacterial activity has also previously been reported with other derived bovine *NK-lysin* peptides [41]. All of these findings suggest that the bovine *NK-lysins* are potentially important in host resistance to respiratory infections.

The inevitable large animal-to-animal variation within animals challenged with the same pathogen in the challenge study may be attributed to individual immunity, which at least partly results from genetic variation, such as gene copy number variations, single nucleotide variants and insertions and deletions. It will be important to investigate variation within members of the bovine *NK-lysin* gene family and their regulatory regions to identify potential associations with host disease phenotypes. For example, the absence of the bovine *NK2B* gene in some Holstein cattle has been revealed in an ongoing study (unpublished data). It will be important to test whether this deletion affects host responses to specific pathogens. Copy number variation of other *NK-lysin* genes should also be tested within and between breeds of cattle. Further studies are also suggested to investigate point mutations especially the nonsynonymous substitutions in the region coding for the functional helices 2 and 3. The extent of genetic variation in the bovine *NK-lysin* gene family is still unknown but its evolutionary history and diversification of function make it an excellent candidate source of variation for application to breeding protocols.

## References

[1] Zasloff M. Antimicrobial peptides of multicellular organisms. Nature. 2002;415(6870):389–95. Epub 2002/01/25. 10.1038/415389a . 11807545

[2] Brogden KA. Antimicrobial peptides: pore formers or metabolic inhibitors in bacteria? Nat Rev Microbiol. 2005;3(3):238–50. Epub 2005/02/11. 10.1038/nrmicro1098 . 15703760

[3] Norrby SR, Nord CE, Finch R. Lack of development of new antimicrobial drugs: a potential serious threat to public health. Lancet Infect Dis. 2005;5(2):115–9. Epub 2005/02/01. 10.1016/s1473-3099(05)01283-1 . 15680781

[4] Pena SV, Hanson DA, Carr BA, Goralski TJ, Krensky AM. Processing, subcellular localization, and function of 519 (granulysin), a human late T cell activation molecule with homology to small, lytic, granule proteins. Journal of immunology (Baltimore, Md: 1950). 1997;158(6):2680–8. Epub 1997/03/15. . 9058801

[5] Andersson M, Gunne H, Agerberth B, Boman A, Bergman T, Sillard R, et al NK-lysin, a novel effector peptide of cytotoxic T and NK cells. Structure and cDNA cloning of the porcine form, induction by interleukin 2, antibacterial and antitumour activity. The EMBO journal. 1995;14(8):1615–25. Epub 1995/04/18. ; PubMed Central PMCID: PMCPmc398254. 7737114 10.1002/j.1460-2075.1995.tb07150.x PMC398254

[6] Linde CM, Grundstrom S, Nordling E, Refai E, Brennan PJ, Andersson M. Conserved structure and function in the granulysin and NK-lysin peptide family. Infect Immun. 2005;73(10):6332–9. Epub 2005/09/24. 10.1128/iai.73.10.6332-6339.2005 16177304 PMC1230960

[7] Jacobs T, Bruhn H, Gaworski I, Fleischer B, Leippe M. NK-lysin and its shortened analog NK-2 exhibit potent activities against *Trypanosoma cruzi*. Antimicrobial agents and chemotherapy. 2003;47(2):607–13. Epub 2003/01/25. ; PubMed Central PMCID: PMCPmc151766. 12543667 10.1128/AAC.47.2.607-613.2003 PMC151766

[8] Yan JX, Wang KR, Chen R, Song JJ, Zhang BZ, Dang W, et al Membrane active antitumor activity of NK-18, a mammalian NK-lysin-derived cationic antimicrobial peptide. Biochimie. 2012;94(1):184–91. Epub 2011/11/01. 10.1016/j.biochi.2011.10.005 . 22037375

[9] Wang Z, Choice E, Kaspar A, Hanson D, Okada S, Lyu SC, et al Bactericidal and tumoricidal activities of synthetic peptides derived from granulysin. Journal of immunology (Baltimore, Md: 1950). 2000;165(3):1486–90. Epub 2000/07/21. . 10903754 10.4049/jimmunol.165.3.1486

[10] Krensky AM. Granulysin: a novel antimicrobial peptide of cytolytic T lymphocytes and natural killer cells. Biochem Pharmacol. 2000;59(4):317–20. Epub 2000/01/22. . 10644038 10.1016/s0006-2952(99)00177-x

[11] Andreu D, Carreno C, Linde C, Boman HG, Andersson M. Identification of an anti-mycobacterial domain in NK-lysin and granulysin. Biochem J. 1999;344 Pt 3:845–9. Epub 1999/12/10. 10585872 PMC1220707

[12] Stenger S, Hanson DA, Teitelbaum R, Dewan P, Niazi KR, Froelich CJ, et al An antimicrobial activity of cytolytic T cells mediated by granulysin. Science (New York, NY). 1998;282(5386):121–5. Epub 1998/10/02. . 9756476 10.1126/science.282.5386.121

[13] Chen J, Buckley R, Malig M, Lawhon SD, Skow L, Lee MO, et al Bovine NK-lysin: copy number variation and functional diversification Proceedings of the National Academy of Sciences of the United States of America. 2015. Epub In press. 10.1073/pnas.1519374113 PMC4702975 26668394

[14] Griffin D. Economic impact associated with respiratory disease in beef cattle. Vet Clin North Am Food Anim Pract. 1997;13(3):367–77. Epub 1997/11/22. . 9368983 10.1016/s0749-0720(15)30302-9

[15] Snowder GD, Van Vleck LD, Cundiff LV, Bennett GL, Koohmaraie M, Dikeman ME. Bovine respiratory disease in feedlot cattle: phenotypic, environmental, and genetic correlations with growth, carcass, and longissimus muscle palatability traits. J Anim Sci. 2007;85(8):1885–92. Epub 2007/05/17. 10.2527/jas.2007-0008 . 17504959

[16] Garcia MD, Thallman RM, Wheeler TL, Shackelford SD, Casas E. Effect of bovine respiratory disease and overall pathogenic disease incidence on carcass traits. J Anim Sci. 2010;88(2):491–6. Epub 2009/11/10. 10.2527/jas.2009-1874 . 19897630

[17] Taylor JD, Fulton RW, Lehenbauer TW, Step DL, Confer AW. The epidemiology of bovine respiratory disease: What is the evidence for predisposing factors? Can Vet J. 2010;51(10):1095–102. Epub 2011/01/05. 21197200 PMC2942046

[18] Muggli-Cockett NE, Cundiff LV, Gregory KE. Genetic analysis of bovine respiratory disease in beef calves during the first year of life. J Anim Sci. 1992;70(7):2013–9. Epub 1992/07/01. . 1644673 10.2527/1992.7072013x

[19] Neibergs H, Zanella R, Casas E, Snowder GD, Wenz J, Neibergs JS, et al Loci on Bos taurus chromosome 2 and Bos taurus chromosome 26 are linked with bovine respiratory disease and associated with persistent infection of *bovine viral diarrhea virus*. J Anim Sci. 2011;89(4):907–15. Epub 2010/12/15. 10.2527/jas.2010-3330 . 21148784

[20] Cernicchiaro N, Renter DG, White BJ, Babcock AH, Fox JT. Associations between weather conditions during the first 45 days after feedlot arrival and daily respiratory disease risks in autumn-placed feeder cattle in the United States. J Anim Sci. 2012;90(4):1328–37. Epub 2011/12/08. 10.2527/jas.2011-4657 . 22147486

[21] Cernicchiaro N, White BJ, Renter DG, Babcock AH, Kelly L, Slattery R. Associations between the distance traveled from sale barns to commercial feedlots in the United States and overall performance, risk of respiratory disease, and cumulative mortality in feeder cattle during 1997 to 2009. J Anim Sci. 2012;90(6):1929–39. Epub 2012/01/17. 10.2527/jas.2011-4599 . 22247119

[22] Snowder GD, Van Vleck LD, Cundiff LV, Bennett GL. Bovine respiratory disease in feedlot cattle: environmental, genetic, and economic factors. J Anim Sci. 2006;84(8):1999–2008. Epub 2006/07/26. 10.2527/jas.2006-046 . 16864858

[23] Welsh RD, Dye LB, Payton ME, Confer AW. Isolation and antimicrobial susceptibilities of bacterial pathogens from bovine pneumonia: 1994–2002. J Vet Diagn Invest. 2004;16(5):426–31. Epub 2004/10/06. . 15460326 10.1177/104063870401600510

[24] Rice JA, Carrasco-Medina L, Hodgins DC, Shewen PE. *Mannheimia haemolytica* and bovine respiratory disease. Anim Health Res Rev. 2007;8(2):117–28. Epub 2008/01/26. 10.1017/s1466252307001375 . 18218156

[25] Shahriar FM, Clark EG, Janzen E, West K, Wobeser G. Coinfection with *bovine viral diarrhea virus* and *Mycoplasma bovis* in feedlot cattle with chronic pneumonia. Can Vet J. 2002;43(11):863–8. Epub 2002/12/25. 12497963 PMC339759

[26] Klima CL, Zaheer R, Cook SR, Booker CW, Hendrick S, Alexander TW, et al Pathogens of bovine respiratory disease in North American feedlots conferring multidrug resistance via integrative conjugative elements. J Clin Microbiol. 2014;52(2):438–48. Epub 2014/01/31. 10.1128/jcm.02485-13 24478472 PMC3911356

[27] Gagea MI, Bateman KG, van Dreumel T, McEwen BJ, Carman S, Archambault M, et al Diseases and pathogens associated with mortality in Ontario beef feedlots. J Vet Diagn Invest. 2006;18(1):18–28. Epub 2006/03/29. . 16566254 10.1177/104063870601800104

[28] Ellis JA. Bovine parainfluenza-3 virus. Vet Clin North Am Food Anim Pract. 2010;26(3):575–93. Epub 2010/11/09. 10.1016/j.cvfa.2010.08.002 . 21056802

[29] Neibergs HL, Seabury CM, Wojtowicz AJ, Wang Z, Scraggs E, Kiser J, et al Susceptibility loci revealed for bovine respiratory disease complex in pre-weaned holstein calves. BMC genomics. 2014;15(1):1164 Epub 2014/12/24. 10.1186/1471-2164-15-1164 . 25534905 PMC4445561

[30] Tizioto PC, Kim J, Seabury CM, Schnabel RD, Gershwin LJ, Van Eenennaam AL, et al Immunological Response to Single Pathogen Challenge with Agents of the Bovine Respiratory Disease Complex: An RNA-Sequence Analysis of the Bronchial Lymph Node Transcriptome. PloS one. 2015;10(6):e0131459 Epub 2015/06/30. 10.1371/journal.pone.0131459 26121276 PMC4484807

[31] Gershwin LJ, Van Eenennaam AL, Anderson ML, McEligot HA, Shao MX, Toaff-Rosenstein R, et al Single Pathogen Challenge with Agents of the Bovine Respiratory Disease Complex. PloS one. 2015;10(11):e0142479 Epub 2015/11/17. 10.1371/journal.pone.0142479 26571015 PMC4646450

[32] Yeaman MR, Yount NY. Mechanisms of antimicrobial peptide action and resistance. Pharmacological reviews. 2003;55(1):27–55. Epub 2003/03/05. 10.1124/pr.55.1.2 . 12615953

[33] Langmead B, Salzberg SL. Fast gapped-read alignment with Bowtie 2. Nature methods. 9(4):357–9. 10.1038/nmeth.1923 ; PubMed Central PMCID: PMCPmc3322381. 22388286 PMC3322381

[34] Chen PS, Toribara TY, Warner H. Microdetermination of Phosphorus. Analytical Chemistry. 1956;28(11):1756–8. 10.1021/ac60119a033

[35] Lee MO, Kim EH, Jang HJ, Park MN, Woo HJ, Han JY, et al Effects of a single nucleotide polymorphism in the chicken NK-lysin gene on antimicrobial activity and cytotoxicity of cancer cells. Proceedings of the National Academy of Sciences of the United States of America. 2012;109(30):12087–92. Epub 2012/07/12. 10.1073/pnas.1209161109 22783018 PMC3409721

[36] Chen Y, Guarnieri MT, Vasil AI, Vasil ML, Mant CT, Hodges RS. Role of peptide hydrophobicity in the mechanism of action of alpha-helical antimicrobial peptides. Antimicrob Agents Chemother. 2007;51(4):1398–406. Epub 2006/12/13. 10.1128/aac.00925-06 17158938 PMC1855469

[37] Glass EJ, Baxter R, Leach RJ, Jann OC. Genes controlling vaccine responses and disease resistance to respiratory viral pathogens in cattle. Veterinary immunology and immunopathology. 2012;148(1–2):90–9. Epub 2011/05/31. 10.1016/j.vetimm.2011.05.009 21621277 PMC3413884

[38] Casas E, Hessman BE, Keele JW, Ridpath JF. A genome-wide association study for the incidence of persistent bovine viral diarrhea virus infection in cattle. Animal genetics. 2015;46(1):8–15. Epub 2014/11/14. 10.1111/age.12239 . 25394207

[39] Bermingham ML, Bishop SC, Woolliams JA, Pong-Wong R, Allen AR, McBride SH, et al Genome-wide association study identifies novel loci associated with resistance to bovine tuberculosis. Heredity (Edinb). 2014;112(5):543–51. Epub 2014/02/06. 10.1038/hdy.2013.137 24496092 PMC3998787

[40] Jongstra J, Schall TJ, Dyer BJ, Clayberger C, Jorgensen J, Davis MM, et al The isolation and sequence of a novel gene from a human functional T cell line. J Exp Med. 1987;165(3):601–14. Epub 1987/03/01. 2434598 10.1084/jem.165.3.601 PMC2188281

[41] Endsley JJ, Furrer JL, Endsley MA, McIntosh MA, Maue AC, Waters WR, et al Characterization of bovine homologues of granulysin and NK-lysin. Journal of immunology (Baltimore, Md: 1950). 2004;173(4):2607–14. Epub 2004/08/06. . 15294977 10.4049/jimmunol.173.4.2607

